# Unsuccessful Uterine Artery Embolization Requiring Subtotal Hysterectomy: A Case Report

**DOI:** 10.7759/cureus.20210

**Published:** 2021-12-06

**Authors:** Emmanuel Kontomanolis, Chrysovalantis Stylianou, Styliani Mitropoulou, Vasileios Balomenos, Vasileios Souftas

**Affiliations:** 1 Obstetrics and Gynecology, University Hospital of Alexandroupolis, Alexandroupolis, GRC; 2 Radiology and Interventional Radiology, University Hospital of Alexandroupolis, Alexandroupolis, GRC

**Keywords:** female reproductive health, female fertility, surgery, hysterectomy, uterine artery embolization (uae), uterine fibroids, leiomyomas

## Abstract

Introduction: The purpose of this case report is to present the case of a 49-year-old female individual with uterine fibroids, who underwent a subtotal hysterectomy after prior unsuccessful uterine artery embolization. Uterine artery embolization is a minimally-invasive technique used as an additional option for the treatment of symptomatic fibroids. The method is a promising technique, indicated for female individuals of reproductive age wishing to preserve their uteri.

Case Presentation: The patient presented symptoms of bloating and menorrhagia. Magnetic Resonance Imaging revealed uterine enlargement and elongation, with several fibroids and urinary bladder deformation caused by the enlarged uterus and the numerous fibroids compressing the uterine apex. She was referred for uterine artery embolization. In the three-year follow-up imaging, no alteration of the uterus’ size was observed, while two fibroids were noted, suggesting necrosis or malignancy based on imaging findings, which led to the conduction of partial hysterectomy while leaving the cervix intact.

Discussion: Uterine artery embolization is regarded as a safe and successful procedure. However, in case of ineffectiveness, full or partial hysterectomy is considered as the ultimate treatment of choice.

Conclusion: According to the available literature, uterine artery embolization is promoted to be an efficient alternative option to surgery. Patients should be offered adequate medical consultation on all the treatment options and possible complications.

## Introduction

Uterine artery embolization (UAE) is a minimally-invasive, radiological arteriographic technique used for treating symptomatic uterine fibroids, also known as uterine leiomyomas [1]. UAE has been promoted as an alternative option to medication, hysterectomy, and myomectomy [2]. In 2008 the American College of Obstetrics and Gynaecology proposed this minimally invasive technique as an effective and safe alternative treatment aiming at female individuals who wish to retain their uteri [3]. UAE mainly focuses on reducing the size of fibroids and on treating abnormal bleeding by occluding the vascular supply of fibroids [3,4]. The procedure can be performed through a single or bilateral femoral approach. Subsequently, the uterine arteries are catheterised under fluoroscopic guidance, and embolic agents are injected, resulting in embolization [4]. However, hysterectomies are also performed mainly to resolve UAE complications [5]. Especially in cases of women of reproductive age, myomectomy remains the main option as there are not sufficient data evaluating complications related to pregnancy [6].

## Case presentation

**Figure 1:**
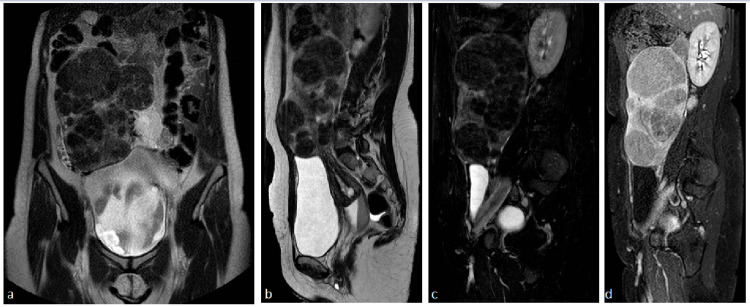
Preprocedural MRI images a) Coronal T2-weighted image shows an enlarged and elongated uterus body (222 mm x 163 mm), with numerous focal lesions demonstrating low signal. Deformation of the urine bladder can be observed. b) Sagittal T2-weighted image in the midline shows the cranial elongation of the uterine corpus, the cervix, and vagina. Nabothian cysts can be seen in the cervix. c) Sagittal T2-weighted fat-saturated image shows the maximum craniocaudal length (222 mm) of the uterus, which was measured laterally towards the right upper quadrant of the abdomen. d) Sagittal T1-weighted, contrast-enhanced, fat-saturated image shows enhancement of all the fibroids.

A 49-year-old female was referred to our interventional radiology department complaining about symptoms of abdominal bloating. The patient was experiencing repeating episodes of menorrhagia lasting 10-11 days each. Laboratory tests reaffirmed the microcytic hypochromic anaemia already known from the patient’s medical history. Additionally, she underwent a preprocedural MRI exam where T1, T2, diffusion-weighted imaging (DWI), fat saturation, and contrast-enhanced sequences were obtained (Figure 1). 

The uterus appeared enlarged and elongated (222 mm x 163 mm), occupying a vast space of the abdomen and deforming the urinary bladder. The uterine wall had numerous focal lesions, showing low signal in T2 sequences and an intermediate signal in T1 sequences, suggesting fibroids. Both the cervix and the vagina were also elongated cranially. Cystic formations in the cervix were noted, imitating Nabothian cysts. After discussing extensively with the patient on her treatment options, she opted for uterine artery embolization submitting her written informed consent. The latter was following our institution’s ethics committee instructions and preserved the patient’s anonymity.

Uterine artery embolization was performed by accessing the right common femoral artery under conscious sedation and local anaesthesia. The uterine arteries were selectively catheterised and embolised post arteriography. The embolic agents used were 350 μm to 710 μm polyvinyl alcohol (PVA) particles and 700 μm acrylic microspheres. No subsequent complications were observed.

**Figure 2:**
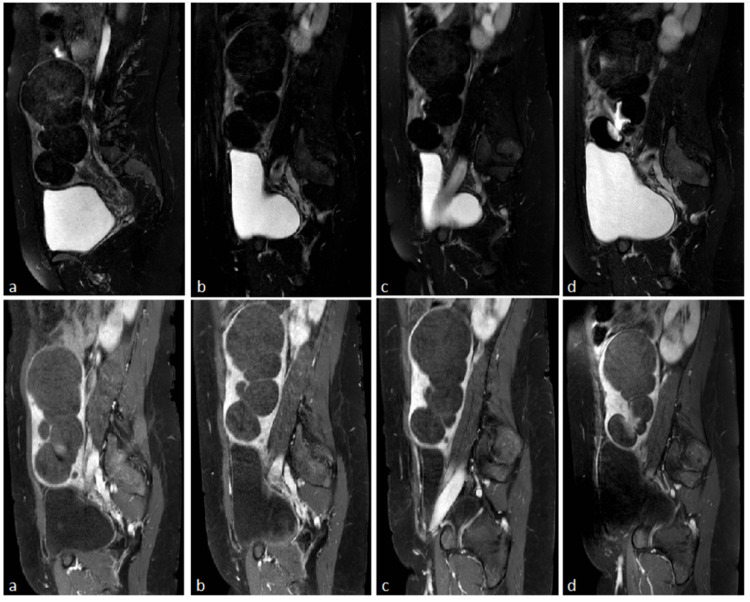
Postprocedural MRI’s Sagittal T2-weighted fat-saturated (up) and T1-weighted, contrast-enhanced, fat-saturated (down) images. a) two months follow-up, confirming significant uterus size reduction (maximum diameter 197 mm) and no enhancement with the contrast agent. b) five months follow-up, with a further reduction to the uterus size (maximum diameter 191 mm) and no enhancement of the fibroids. c) 12 months follow-up, indicating no further uterine size reduction (maximum diameter 190 mm) and unenhanced fibroids. d) 36 months follow-up, with all fibroids reappearing ischemic and in the same size as previously measured. Two submucosal fibroids at the anterior and posterior uterine walls (mirror-like) contained branching tissue infiltrations, displaying normal endometrial tissue.

Our follow-up protocol consists of MRI imaging two, five, 12, and 36 months after the UAE (Figure 2).

During our first follow-up session, a small amount of free intraperitoneal fluid was observed. Both first and second sessions revealed a significant uterus’ size reduction (maximum diameter 197 mm and 191 mm, respectively), and all fibroids were not enhanced with contrast. One-year post-embolization (third follow-up session), the uterus was not further reduced (maximum diameter 190 mm), and the fibroids; though, had small changes in size, remained contrast-unenhanced. Nabothian cysts were observed during all three follow-up sessions. The last follow-up MRI session, 36 months after the UAE, affirmed the uterus’s unaltered status compared to the third follow-up session two years ago. All fibroids appeared ischemic and in the same size as previously measured, with no contrast enhancement. Additionally, two submucosal necrotic fibroids were located at the anterior and posterior uterine walls (mirror-like) containing branching tissue infiltrations. They appeared as normal endometrial tissue in all sequences, with no diffusion in the DWI sequence and normal enhancement. The proposed diagnosis was wedge-shaped spaces of necrotic tissue, which drained in the uterine cavity and then enclosed normal endometrial tissue, and the rare occasion of neoplastic tissue from a stromatic tumor originating from the endometrium. The ovaries appeared normal. A hysterectomy was recommended to the patient as the best treatment option regarding her situation at the time.

After submitting her written consent (the same procedure was followed as in UAE according to the ethics committee instructions), partial hysterectomy (surgical excision of uterus, ovaries, and fallopian tubes leaving the cervix in place) was performed, and samples were received for lab analysis without postoperative complications. Histopathology revealed adenomyosis foci with multiple fibroids and extensive degenerated lesions (calcifications and vitrification). Malignant lesions were not detected. The patient was discharged in stable condition on the second postoperative day after an uneventful course.

## Discussion

We present a case of a 49-year-old female patient who underwent a partial hysterectomy after an unsuccessful UAE for fibroids treatment.

UAE is broadly considered a safe and successful interventional procedure. Studies have suggested that symptoms of menorrhagia and pelvic pain, as well as uterine size, are reduced after a UAE session [6-8]. However, it can fail even when the embolization procedure is uneventful. This can occur mostly as a result of ovarian arteries supplying the uterus and thus the fibroids. Uterine and ovarian arteries should be thoroughly analysed via imaging as they can follow a very complicated course to provide for all portions of the fallopian tubes [9,10]. Taking into consideration that fibroids derive from various areas (fallopian tube, broad ligament, etc.), pre-treatment MRI can be a useful tool in assessing the success of UAE [11].

In the case of unsuccessful UAE, total or subtotal hysterectomy is considered as the treatment of choice, although a rather rare scenario [2,10]. It is very important to mention the fact that UAE includes a series of adverse outcomes which eventually could lead to infertility, and thus the patient should be extensively informed by the physician performing the procedures (interventional or surgical). In our case, the patient opted for the UAE to avoid surgery. However, as UAE was unsuccessful, surgical resection was the only treatment of choice.

## Conclusions

It is of utter importance that female patients are offered adequate medical consultation on all the treatment options (UAE, hysterectomy, myomectomy, medication) for the most suitable treatment to be decided. Uterine anatomy and vascular supply, the location, the number and the size of the fibroids, as well as the patient’s age and desire for future pregnancy, are factors that should be taken into consideration in choosing the proper treatment. In addition, female patients should also be extensively informed about the possible complications and failure possibilities of each treatment choice. In case of an unsuccessful UAE procedure partial, or total hysterectomy for the management of symptomatic fibroids is regarded as the treatment of choice.

## References

[1] Ravina JH, Herbreteau D, Ciraru-Vigneron N (1995). Arterial embolisation to treat uterine myomata. Lancet.

[2] Yeagley TJ, Goldberg J, Klein TA, Bonn J (2002). Labial necrosis after uterine artery embolization for leiomyomata. Obstet Gynecol.

[3] Felemban A, Stein L, Tulandi T (2001). Uterine restoration after repeated expulsion of myomas after uterine artery embolization. J Am Assoc Gynecol Laparosc.

[4] Spies JB (2016). Current role of uterine artery embolization in the management of uterine fibroids. Clin Obstet Gynecol.

[5] Toor SS, Jaberi A, Macdonald DB, McInnes MD, Schweitzer ME, Rasuli P (2012). Complication rates and effectiveness of uterine artery embolization in the treatment of symptomatic leiomyomas: a systematic review and meta-analysis. Am J Roentg.

[6] Hurst BS, Stackhouse DJ, Matthews ML, Marshburn PB (2000). Uterine artery embolization for symptomatic uterine myomas. Fertil Steril.

[7] Amato P, Roberts AC (2001). Transient ovarian failure: a complication of uterine artery embolization. Fertil Steril.

[8] Pron G, Cohen M, Soucie J (2003). The Ontario uterine fibroid embolization Trial. Part 1. Baseline patient characteristics, fibroid burden, and impact on life. Fertil Steril.

[9] Farrer-Brown G, Beilby JO, Tarbit MH (1970). The blood supply of the uterus. 1. Arterial vasculature. J Obstet Gynaecol Br Commonw.

[10] Stringer NH, Grant T, Park J, Oldham L (2000). Ovarian failure after uterine artery embolization for treatment of myomas. J Am Assoc Gynecol Laparosc.

[11] Jha RC, Ascher SM, Imaoka I, Spies JB (2000). Symptomatic fibroleiomyomata: MR imaging of the uterus before and after uterine arterial embolization. Radiology.

